# Neurological Evidence Linguistic Processes Precede Perceptual Simulation in Conceptual Processing

**DOI:** 10.3389/fpsyg.2012.00385

**Published:** 2012-10-16

**Authors:** Max Louwerse, Sterling Hutchinson

**Affiliations:** ^1^Department of Psychology, Institute for Intelligent Systems, University of MemphisMemphis, TN, USA

**Keywords:** embodied cognition, symbolic cognition, symbol interdependency, perceptual simulation, language processing, EEG

## Abstract

There is increasing evidence from response time experiments that language statistics and perceptual simulations both play a role in conceptual processing. In an EEG experiment we compared neural activity in cortical regions commonly associated with linguistic processing and visual perceptual processing to determine to what extent symbolic and embodied accounts of cognition applied. Participants were asked to determine the semantic relationship of word pairs (e.g., *sky – ground*) or to determine their iconic relationship (i.e., if the presentation of the pair matched their expected physical relationship). A linguistic bias was found toward the semantic judgment task and a perceptual bias was found toward the iconicity judgment task. More importantly, conceptual processing involved activation in brain regions associated with both linguistic and perceptual processes. When comparing the relative activation of linguistic cortical regions with perceptual cortical regions, the effect sizes for linguistic cortical regions were larger than those for the perceptual cortical regions early in a trial with the reverse being true later in a trial. These results map upon findings from other experimental literature and provide further evidence that processing of concept words relies both on language statistics and on perceptual simulations, whereby linguistic processes precede perceptual simulation processes.

## Introduction

Conceptual processing elicits perceptual simulations. For instance, when people read the word pair *sky – ground*, one word presented above the other, processing is faster when *sky* appears above *ground* than when the words are presented in the reversed order (Zwaan and Yaxley, 2003; Louwerse, 2008; Louwerse and Jeuniaux, 2010). Embodiment theorists have interpreted this finding as evidence that perceptual and biomechanical processes underlie cognition (Glenberg, 1997; Barsalou, 1999). Indeed, numerous studies show that processing is affected by tasks that invoke the consideration of perceptual features (see Pecher and Zwaan, 2005; De Vega et al., 2008; Semin and Smith, 2008; for overviews). Much of this evidence comes from behavioral response time (RT) experiments, but there is also evidence stemming from neuropsychological studies (Buccino et al., 2005; Kan et al., 2003; Rueschemeyer et al., 2010). This embodied cognition account is oftentimes presented in contrast to a symbolic cognition account that suggests conceptual representations are formed from statistical linguistic frequencies (Landauer and Dumais, 1997). Such a symbolic cognition account that uses the mind-as-a-computer metaphor has occasionally been dismissed by embodiment theorists (Van Dantzig et al., 2008).

Recently, researchers have cautioned pitting one account against another, demonstrating that symbolic and embodied cognition accounts can be integrated (Barsalou et al., 2008; Louwerse, 2008, 2011; Simmons et al., 2008). For instance, Louwerse (2011) proposed the Symbol Interdependency Hypothesis, arguing that language encodes embodied relations which language users can use as a shortcut during conceptual processing. The relative importance of language statistics and perceptual simulation in conceptual processing depends on several variables, including the type of stimulus presented to a participant, and the cognitive task the participant is asked to perform (Louwerse and Jeuniaux, 2010). Louwerse and Connell (2011) further found that the effects for language statistics on processing times temporally preceded the effects of perceptual simulations on processing times, with fuzzy regularities in linguistic context being used for quick decisions and precise perceptual simulations being used for slower decisions. Importantly, these studies do not deny the importance of perceptual processes. In fact, individual effects for perceptual simulations were also seen early on in a trial, however, when comparing the effect sizes of language statistics and perceptual simulations, Louwerse and Connell (2011) found evidence for early linguistic and late perceptual simulation processes.

The results from these RT studies, however, only indirectly demonstrate that language statistics and perceptual simulation are active during cognition, because the effects are modulated by hand movements and RTs. Although such methods are methodologically valid, we sought to establish whether such conclusions were also supported by neurological evidence.

In the current paper our objective was to determine when conceptual processing uses neurological processes best explained by language statistics relative to neurological processes best explained by perceptual simulations. Given the evidence that both statistical linguistic frequencies and perceptual simulation are involved in conceptual processing (Louwerse, 2008; Simmons et al., 2008; Louwerse and Jeuniaux, 2010), and that the effect for language statistics outperforms the effect for perceptual simulations for fast RTs, with the opposite being true for slower RTs (Louwerse and Connell, 2011), we predicted that cortical regions commonly associated with linguistic processing, when compared with activation in cortical regions commonly associated with perceptual simulation, would be activated relatively early in a RT trial. Conversely, when compared with activation in cortical regions commonly associated with linguistic processing, cortical regions associated with perceptual simulation were predicted to show greater activity relatively later in a RT trial. Further, we predicted activation would be modified by the cognitive task, such that perceptual cortical regions would be more active in a perceptual simulation task, whereas linguistic cortical regions would be more active in a semantic judgment task.

Traditional EEG methodologies are not quite sufficient to answer this research question. For instance, event-related potential (ERP) methods only allow for analyses of time-locked components that activate in response to specific events over numerous trials (Collins et al., 2011; Hald et al., 2011). EEG recordings combined with magnetoencephalography (MEG) recordings can provide high-resolution temporal information and spatial estimates of neural activity, provided that appropriate source reconstruction techniques are used (Hauk et al., 2008). However, this technique establishes whether and when cortical regions are activated, but does not answer the question of what cortical regions are activated in relation to each other. Such a comparative analysis seems to call for a different and novel method.

We utilized source localization techniques in conjunction with statistical analyses to determine when and where relative effects of linguistic and perceptual processes occurred. We did this by investigating which regions of the cortex are responsible for activity throughout the time course of each trial. However, source localization determines only where differences emerge between conditions at specific points in time; our goal was to determine whether relatively stronger early effects of linguistic processes preceded a relatively stronger later simulation process. Consequently, we used established source localization techniques (Pascual-Marqui, 2002) to determine where differences in activation were present during an early versus a late time period. With that information we then ran a mixed effects model on electrode activation throughout the duration of a trial to identify the effect size for activation of linguistic versus perceptual cortical regions over time. This type of analysis is progressive in that it allowed us not only to determine that activation differed between linguistic and perceptual cortical regions but also allowed us to gain insight into the relative effect size of language statistics and perceptual simulation as they contribute to conceptual processing throughout the time course of a trial.

## Materials and Methods

### Participants

Thirty-three University of Memphis undergraduate students participated for extra credit in a psychology course. All participants had normal or corrected vision and were native English speakers. Fifteen participants were randomly assigned to the semantic judgment condition, and 18 participants were randomly assigned to the iconicity judgment condition.

### Materials

Each condition consisted of 64 iconic/reverse-iconic word pairs extracted from previous research (Louwerse, 2008; Louwerse and Jeuniaux, 2010; see Appendix). Thirty-two pairs with an iconic relationship were presented vertically on the screen in the same order they would appear in the world (i.e., *sky* appears above *ground*). Likewise, 32 pairs with a reverse-iconic relationship appeared in an order opposite of that which would be expected in the world (i.e., *ground* appears above *sky*). The remaining 128 trials contained filler word pairs that had no iconic relationship. Half of the fillers had a high semantic relation (cos = 0.55) and half had a low semantic relation (cos = 0.21), as determined by latent semantic analysis (LSA), a statistical, corpus-based, technique for estimating semantic similarities on a scale of −1 to 1 (Landauer et al., 2007). All items were counterbalanced such that all participants saw all word pairs, but no participant saw the same word pair in both orders (i.e., both the iconic and the reverse-iconic order for the experimental items).

### Equipment

An Emotiv EPOC headset (Emotiv Systems Inc., San Francisco, CA, USA) was used to record electroencephalograph data. EEG data recorded from the Emotiv EPOC headset is comparable to data recorded by traditional EEG devices (Bobrov et al., 2011; Stytsenko et al., 2011). For instance, patterns of brain activity from a study in which participants imagined pictures were comparable between the 16-channel Emotiv EPOC system and the 32-channel ActiCap system (Brain Products, Munich, Germany; Bobrov et al., 2011). The Emotiv EPOC is also able to reliably capture P300 signals (Ramírez-Cortes et al., 2010; Duvinage et al., 2012), even though the accuracy of high-end systems is superior.

The headset was fitted with 14 Au-plated contact-grade hardened BeCu felt-tipped electrodes that were saturated in a saline solution. Although the headset used a dry electrode system, such technology has shown to be comparable to traditional wet electrode systems (Estepp et al., 2009). The headset used sequential sampling at 2048 Hz and was down-sampled to 128 Hz. The incoming signal was automatically notch filtered at 50 and 60 Hz using a 5th order sinc notch filter. The resolution was 1.95 μV.

### Procedure

In both the semantic judgment and iconicity judgment conditions, word pairs were presented vertically on an 800 × 600 computer screen. In the semantic judgment condition, participants were asked to determine whether a word pair was related in meaning. In the iconicity judgment condition, participants were asked whether a word pair appeared in an iconic relationship (i.e., if a word pair appeared in the same configuration as the pair would occur in the world). Participants responded to stimuli by pressing designated yes or no keys on a number pad. Participants were instructed to move and blink as little as possible. Word pairs were randomly presented for each participant in order to negate any order effects. To ensure participants understood the task, a session of five practice trials preceded the experimental session.

## Results

We followed prior research (Louwerse, 2008; Louwerse and Jeuniaux, 2010) in identifying errors and outliers. As in those studies, error rates were expected to be high in both the semantic judgment task and the iconicity task. Although some word pairs may share a low semantic relation according to LSA, sometimes for at least one word meaning, a higher semantic relationship might be warranted (see Louwerse et al., 2006). For example, according to LSA, *rib* and *spinach* has a low semantic relation (cos = 0.07), but in one meaning of *rib* (that of barbecue) such a low semantic relation is not justified (Louwerse and Jeuniaux, 2010). For the semantic judgment task, error rates were unsurprisingly approximately 25% (*M* = 26.07, *SD* = 7.51). Similarly, for the iconicity judgment condition, error performance can also be explained by the task. *Priest* and *flag* are not assumed to have an iconic relation, even though such a relation could be imagined. Error rates were around 25–30% (*M* = 29, *SD* = 8.53). For both the semantic judgment condition and the iconicity judgment condition, these error rates were comparable with those reported elsewhere (Louwerse and Jeuniaux, 2010). Analyses of the errors revealed no evidence for a speed-accuracy trade-off. In the RT analysis, data from each subject whose RTs fell more than 2.5 *SD* from the mean per condition, per subject, were removed from the analysis, affecting less than 3% of the data in both experiments.

A mixed effects regression analysis was conducted on RTs with order (*sky* above *ground* or *ground* above *sky*) as a fixed factor and participants and items as random factors (Richter, 2006; Baayen et al., 2008). *F*-test denominator degrees of freedom for RTs were estimated using the Kenward–Roger’s degrees of freedom adjustment to reduce the chances of Type I error (Littell et al., 2002). For the semantic judgment condition, differences were found between the iconic and the reverse-iconic word pairs *F*(1, 2683.75) = 3.7, *p* = 0.05, with iconic word pairs being responded to faster than reverse-iconic word pairs, *M* = 1592.92, SE = 160.46 versus *M* = 1640.06, SE = 159.8. A similar result was obtained for the iconicity judgment condition, *F*(1, 3332.39) = 13.58, *p* < 0.001, again with iconic word pairs being responded to faster than reverse-iconic word pairs, *M* = 1882.87, SE = 155.43 versus *M* = 1980.80, SE = 154.67. This RT advantage has been reported elsewhere (Zwaan and Yaxley, 2003; Louwerse, 2008; Louwerse and Jeuniaux, 2010). What is not clear from these results is whether this effect can be explained by an embodied cognition account (iconicity through perceptual simulations), by a symbolic cognition account (word-order frequency), or by both. As in Louwerse and Jeuniaux (2010) language statistics and perceptual simulations were operationalized using word-order frequency and iconicity ratings.

### Order frequency

Language statistics were operationalized as the log frequency of *a*-*b* (e.g., *sky – ground*) and *b*-*a* (e.g., *ground – sky*) order of word pairs (cf. Louwerse, 2008; Louwerse and Jeuniaux, 2010; Louwerse and Connell, 2011). The order frequency of all 64 word pairs within 3–5 word grams was obtained using the large Web 1T 5-gram corpus (Brants and Franz, 2006).

### Iconicity ratings

Twenty-four participants at the University of Memphis estimated the likelihood that concepts appeared above one another in the real world. Ratings were made for 64 word pairs on a scale of 1–6, with 1 being extremely unlikely and 6 being extremely likely. Each participant saw all word pairs, but whether a participant saw a word pair in an iconic or a reverse iconic order was counterbalanced such that each participant saw iconic and reverse-iconic word pairs, but no participant saw a word pair both in an iconic and a reverse-iconic order. High interrater reliability was found in both groups (Group A: average *r* = 0.76, *p* < 0.001, *n* = 64; Group B: average *r* = 0.74, *p* < 0.001, *n* = 64), with a negative correlation between the two groups (average *r* = −0.72, *p* < 0.001, *n* = 64).

**Figure 1:**
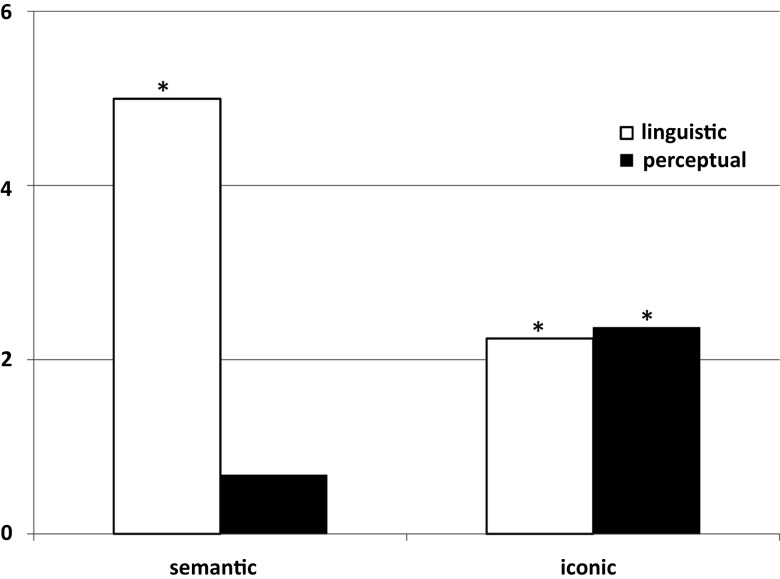
**Strength of the mixed effects regressions on the RTs in absolute *t*-values for each of the two conditions for linguistic (order frequency) and perceptual (iconicity ratings) factors**. Asterisks mark significant strengths (p < 0.05) of relationship with RTs.

A mixed effects regression was conducted on RTs with order frequencies and iconicity ratings as fixed factors and participants and items as random factors. For the semantic judgment condition, a mixed effects regression showed that statistical linguistic frequencies significantly predicted RTs, *F*(1, 760.86) = 24.95, *p* < 0.001, with higher frequencies yielding faster RTs. Iconicity ratings did not yield a significant relation with RT, *F*(1, 762.09) = 0.46, *p* = 0.5 (see the first two bars in Figure 1; Table 1).

**Table 1:** **Regression coefficients for the semantic judgment and iconicity judgment RT experiment**.

	variables	Estimate (SE)	*t* (d*f*)	CI lower	CI upper
Semantic judgment	Intercept	2020.25 (192.11)	10.52 (37.85)**	1631.29	2409.21
	Language statistics	−62.12 (12.44)	−4.99 (760.86)**	−86.54	−37.71
	Iconicity ratings	14.16 (20.97)	0.68 (762.09)	−27.01	55.34
Iconicity judgment	Intercept	2242.95 (185.94)	12.06 (46.41)**	1868.75	2617.15
	Language statistics	−27.50 (12.26)	−2.24 (945.78)*	−51.55	−3.44
	Iconicity ratings	−48.79 (20.60)	−2.37 (947.65)*	−89.21	−8.36

*Note*. Dependent variable is response time; **p < 0.01, *p < 0.05.

### Response times

For the iconicity judgment condition, a mixed effects regression showed statistical linguistic frequencies again significantly predicted RT, *F*(1, 945.78) = 5.03, *p* = 0.03, with higher frequencies yielding faster RTs. Iconicity ratings also yielded a significant relation with RT, *F*(1, 947.65) = 5.61, *p* = 0.02, with higher iconicity ratings yielding lower RTs (see the second two bars in Figure 1; Table 1).

Figure 1 shows that statistical linguistic frequencies explained RTs in both the semantic judgment and the iconicity judgment conditions, but the effect was stronger in the semantic judgment than in the iconicity judgment condition. Figure 1 and Table 1 also show the opposite results for perceptual simulation in that during the semantic judgment condition, the effect of perceptual simulation on RT was limited (and not significant). However, in the iconicity judgment condition, perceptual simulation was significant. The interaction for linguistic frequencies and condition (semantic versus iconic) was significant, *F*(2, 1005.05) = 15.88, *p* < 0.001, as was the interaction for perceptual simulation and condition, *F*(2, 1634.20) = 2.9, *p* = 0.05. Indeed, the overall interaction between factors (linguistic and perceptual) and condition was significant, *F*(2, 1540.18) = 8.10, *p* < 0.001.

These findings replicate the RT data in Louwerse and Jeuniaux (2010). That is, order frequency better explained RTs than the iconicity ratings did in the semantic judgment task, but iconicity ratings better explained RTs than the order frequency did in the iconicity judgment task.

### EEG activation

As discussed earlier, we utilized previously established EEG source localization techniques in conjunction with statistical analyses to determine when and where relative effects of linguistic and perceptual processes occurred. Continuous neural activity was recorded from 14 international 10–20 sites (AF3, F7, F3, FC5, T7, P7, O1, O2, P8, T8, FC6, F4, F8, and AF4; Reilly, 2005, p. 139). Scalp recordings were referenced to CMS/DRL (P3/P4) locations. All electrode impedances were kept below 10 kΩ. As the Emotiv EPOC headset is noisier than high-end systems, to minimize oculomotor, motor, and electrogalvanic artifacts, a high-pass hardware filter removed signals below 0.16 Hz and a low-pass filter removed signals above 30 Hz (see Bobrov et al., 2011 and Duvinage et al., 2012 for similar filtering ranges with the Emotiv EPOC headset). The EEG was sampled at 2048 Hz and was down-sampled to 128 Hz. Gross eye blink and movement artifacts over 150 μV were excluded from the analysis. All data were wirelessly collected via a proprietary Bluetooth USB chip operating in the same frequency range as the headset (2.4 GHz). Data were recorded using Emotiv Testbench software (Emotiv Systems, Inc., San Francisco, CA, USA).

Data were filtered using EEGLAB (Delorme and Makeig, 2004), an open-source toolbox for MATLAB (Mathworks, Inc., Natick, MA, USA). Independent component analyses were implemented using ADJUST, an algorithm that automatically identifies stereotyped temporal and spatial artifacts (Mognon et al., 2010). Any remaining oculomotor or motor activity was visually identified and removed from the dataset.

On average, subjects took 1809 ms to process and respond to the words presented on the screen. Therefore the sLORETA package (Pascual-Marqui, 2002) was used to localize general activity at an early (97–291 ms) and a late (1551–1744 ms) time interval (as we predicted linguistic processes would precede perceptual simulation) in both conditions. The early time period began shortly after presentation of the stimuli and the late time period began shortly before the subject response. LORETA used the MNI152 template (Fuchs et al., 2002) to compute a non-parametric topographical analysis of variance comparing differences between two maps of averaged cortical activity over each time period (Strik et al., 1998). The topographies significantly differed between conditions at early, *p* < 0.01, and late, *p* < 0.01, intervals, with the maximum source for the early time period being found around the left inferior frontal gyrus (iFG; near electrode sites FC5, F7, and T7) and the maximum source for the late time period being found near the lingual gyrus (near electrode sites O1, O2, P7, and P8). As source localization with EEG poorly maps anatomical correlates to function, these sites are obviously approximations of the relevant underlying cortical regions (Nunez and Srinivasan, 2005). Note we are not attempting to pinpoint exact regions of neural activity at a given time but instead we are simply attempting to compare general estimates of neural activity in early versus late processing (i.e., we would like to determine when processing occurs in more linguistic versus in more perceptual regions over the duration of a trial).

Although neural processes are quite distributed and bilaterally activate multiple cortical regions (Bullmore and Sporns, 2009; Bressler and Menon, 2010), there is considerable agreement that specific regions (such as the left iFG and left superior temporal gyrus (STG) consistently show increased activation during language processing (Cabeza and Nyberg, 2000; Papathanassiou, 2000; Blank et al., 2002; De Carli et al., 2007). The same applies to visual perception and visual imagery processes, which bilaterally activate multiple cortical regions, in particular occipital and parietal lobes (Kosslyn et al., 1993, 1999; Alivisatos and Petrides, 1996). Further, visual imagery of words activates these same regions that process incoming perceptual information (Ganis et al., 2004). Reichle et al. (2000) used fMRI to demonstrate that when told to rely on visual imagery while processing linguistic information, subjects were more likely to show increased activation in parietal lobes. As expected, when asked to rely on verbal strategies, activation in traditional language processing regions dominated. Finally, in an fMRI study, Simmons et al. (2008) found that when asked to generate situations in which a word might occur, subjects showed increased activity in the cuneus, precuneus, posterior cingulate gyrus, retrospinal cortex, and lateral parietal cortex. However, when asked to participate in a word association task, activation occurred in language processing regions of the brain, specifically the lateral left iFG and the medial inferior frontal. During early conceptual processing (first 7.5 s of a 15 s trial), activation was similar to that of the word association task (i.e., these same language processing areas were active). This is consistent with our output from sLORETA in that during early processing, the maximum source was also the left iFG. Unlike early processing, Simmons et al. (2008) found that late conceptual processing (last 7.5 s of a 15 s trial) resulted in activation of the precuneus, posterior cingulate gyrus, and the right lateral parietal cortex (regions all closest to electrodes P7, P8, O1, and O2), the same regions active during situation generation. Although our sLORETA source localization indicated that the maximum source for our late time period was near the lingual gyrus, this region is also in closest proximity to electrode sites P7, P8, O1, and O2.

**Figure 2:**
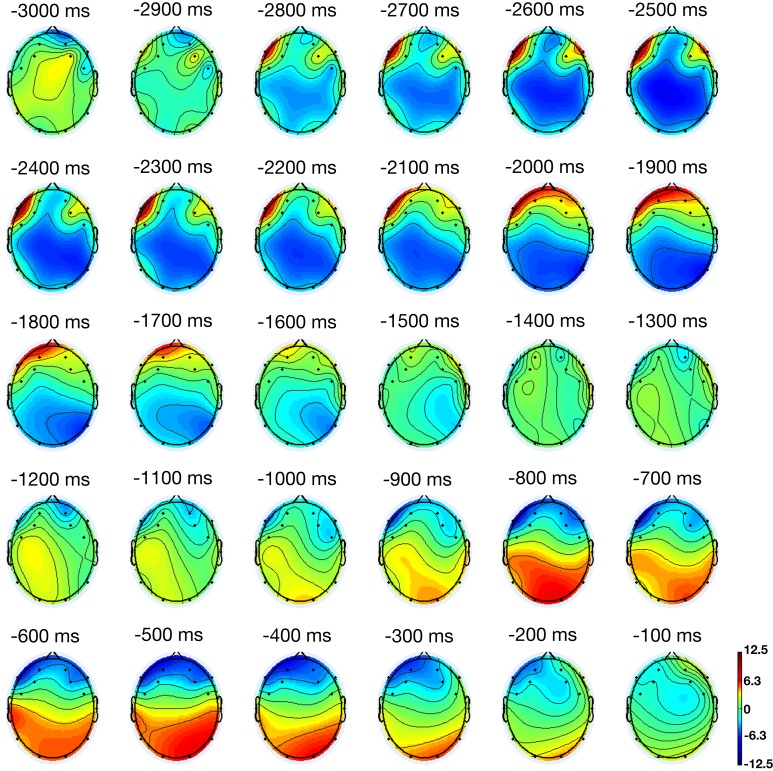
**Cortical activation throughout a trial**. Presentation of the experimental stimulus (i.e., word pair) starts at −2800 ms.

Figure 2 shows the activation for a participant averaged across all trials in 100 ms increments. A relatively localized increase in activation in linguistic processing regions began almost immediately after a stimulus was presented. Around the middle of the trial, the activation dispersed from the linguistic processing regions toward perceptual processing regions. Late in the trial, localized activation was relatively greater in perceptual processing regions. This pattern matches the conclusions drawn by Louwerse and Connell (2011) on the basis of RT data and the results obtained through sLORETA, that linguistic processes precede perceptual processes.

To complement the pattern observed in Figure 2 in both our RT data and in the sLORETA results, we performed a mixed effects regression on electrode activation. We assigned the linguistic cortical regions, as determined by sLORETA localization, a dummy value of 1, and we assigned the perceptual cortical regions, as determined by sLORETA localization, a dummy value of 2. We used electrode activation as our dependent variable, and participant, item, and receptor as random factors. The reason we used individual receptors as random factors was to rule out strong effects that could be observed for one receptor but not for others within the regions commonly associated with linguistic or perceptual processing. With this analysis, our objective was to determine to what extent linguistic or perceptual cortical regions overall showed increased activation throughout the trial. As in the previous analyses, *F*-test denominator degrees of freedom for the dependent variable were estimated using the Kenward–Roger’s degrees of freedom adjustment.

For the semantic judgment condition, a significant difference was observed between linguistic and perceptual cortical regions, *F*(1, 1153108.58) = 46.70, *p* < 0.001. A similar pattern was found for the iconicity judgment condition, *F*(1, 1464148.76) = 24.07, *p* < 0.001. The fact that a difference was observed is perhaps uninteresting; differences between linguistic and perceptual regions are expected. Instead, the direction of the effect is important here. Recall that linguistic regions were dummy coded as 1, and perceptual regions were dummy coded as 2. Positive *t*-values would indicate that perceptual regions dominate, and negative *t*-values would indicate that linguistic regions dominate. Based on the findings in the RT analysis reported above, we predicted that linguistic regions would dominate in both the semantic and iconicity task, and more so in the semantic judgment task than in the iconicity judgment task. This prediction is supported by the results; *t*-values in both the semantic and iconicity tasks were negative, as predicted with higher *t*-values in the semantic task, *t* (1153109) = −6.83, *p* < 0.001, than in the iconicity task, *t* (1464149) = −4.91, *p* < 0.001, replicating the RT findings.

**Figure 3:**
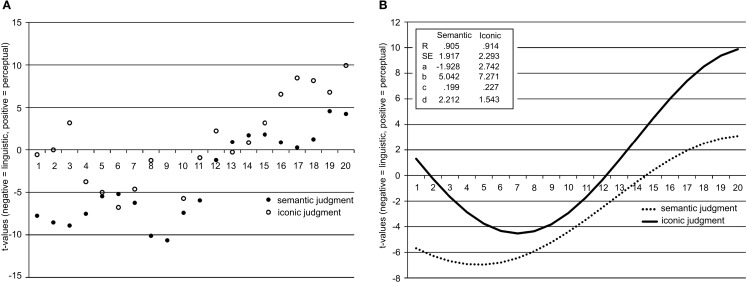
**(A)** *t*-values for each of the 20 time bins for both the semantic judgment and iconicity judgment conditions. Negative *t*-values represent a relative bias toward linguistic cortical regions, positive *t*-values represent a relative bias toward perceptual cortical regions. **(B)** *t*-values for each of the 20 time bins for both the semantic judgment and iconicity judgment conditions fitted using a sinusoidal curve model and correlation coefficients, standard errors, and parameter coefficients for the sinusoidal model, y = a + b × cos (cx + d). Negative *t*-values represent a relative bias toward linguistic cortical regions, positive *t*-values represent a relative bias toward perceptual cortical regions.

To determine whether linguistic processes precede perceptual simulation processes, we created 20 time bins for each trial per participant, per condition (cf. Louwerse and Bangerter, 2010). Each time bin was therefore approximately 80 ms for the semantic judgment condition and 95 ms for the iconicity judgment condition. Twenty time bins allowed for the largest number of groups for examining trends of each factor while retaining sufficient data points per participant to test the time course hypotheses. Mixed effects models were again run, now with time bin as an added predictor in the model. The *t*-values of the mixed effects models per time bin are shown in Figure 3A, Tables 2 and 3. The figure shows that *t*-values in both the semantic judgment and the iconicity judgment experiments are predominantly negative in the first half of the trial (suggesting a bias toward cortical regions associated with linguistic processing), and predominantly positive toward the end of the trial (suggesting a bias toward cortical regions associated with perceptual processing). Note here that these are the relative effect sizes for the two clusters of cortical regions (FC5, F7, and T7) and (O1, O2, P7, and P8), with the effects for individual electrodes filtered out. The findings do not show low activation for the perceptual processing areas early on in the trial (as words must of course be recognized by the visual system during processing); these results merely show that, relative to the brain regions associated with linguistic processing, the effect sizes of perceptual processing regions dominate later in the trial. Also note the relative effect for brain regions associated with perceptual processing very early in the trial (time bins 1–4), perhaps in line with the early activation of perceptual simulations (Hauk et al., 2008; Pulvermüller et al., 2009).

**Table 2:** **Regression coefficients semantic judgment task EEG experiment**.

Time bin	variables	Estimate (SE)	*t* (d*f*)	CI lower	CI upper
1	Intercept	−1.14 (2.45)	−0.47 (20.77)	−6.23	3.95
	Ling.-perc. bias	−4.71 (0.73)	−6.41 (66071.42)**	−6.15	−3.27
2	Intercept	−1.30 (2.51)	−0.52 (21.64)	−6.51	3.90
	Ling.-perc. bias	−5.34 (0.76)	−7.04 (65935.04)**	−6.83	−3.85
3	Intercept	0.34 (2.85)	0.12 (37.32)	−5.44	6.11
	Ling.-perc. bias	−5.52 (0.75)	−7.36 (66088.74)**	−6.99	−4.05
4	Intercept	0.19 (1.93)	0.10 (25.32)	−3.79	4.16
	Ling.-perc. bias	−4.40 (0.71)	−6.21 (65996.83)**	−5.79	−3.01
5	Intercept	−0.29 (1.44)	−0.20 (27.58)	−3.25	2.67
	Ling.-perc. bias	−3.07 (0.69)	−4.45 (65100.18)**	−4.42	−1.72
6	Intercept	−0.99 (1.37)	−0.72 (32.15)	−3.78	1.80
	Ling.-perc. bias	−3.00 (0.69)	−4.35 (66873.74)**	−4.35	−1.65
7	Intercept	0.39 (1.33)	0.29 (31.12)	−2.32	3.11
	Ling.-perc. bias	−3.53 (0.68)	−5.16 (66148.30)**	−4.87	−2.19
8	Intercept	2.89 (1.22)	2.36 (32.04)*	0.40	5.38
	Ling.-perc. bias	−5.69 (0.68)	−8.39 (66364.66)**	−7.02	−4.36
9	Intercept	2.96 (1.04)	2.84 (43.87)**	0.86	5.06
	Ling.-perc. bias	−5.85 (0.67)	−8.78 (65944.93)**	−7.16	−4.54
10	Intercept	2.50 (1.21)	2.07 (31.56)*	0.04	4.97
	Ling.-perc. bias	−4.07 (0.67)	−6.03 (65120.78)**	−5.39	−2.74
11	Intercept	2.29 (1.26)	1.82 (31.59)	−0.28	4.86
	Ling.-perc. bias	−3.42 (0.68)	−5.04 (67761.30)**	−4.75	−2.09
12	Intercept	0.10 (1.13)	0.09 (32.78)	−2.20	2.40
	Ling.-perc. bias	−0.69 (0.69)	−1.00 (65801.13)	−2.05	0.67
13	Intercept	−0.43 (1.14)	−0.38 (30.18)	−2.77	1.90
	Ling.-perc. bias	0.52 (0.67)	0.77 (66336.68)	−0.80	1.84
14	Intercept	0.32 (1.00)	0.32 (48.81)	−1.69	2.33
	Ling.-perc. bias	0.96 (0.68)	1.43 (66148.68)	−0.36	2.29
15	Intercept	1.45 (1.22)	1.19 (35.46)	−1.02	3.92
	Ling.-perc. bias	0.98 (0.65)	1.53 (66886.19)	−0.28	2.25
16	Intercept	2.21 (1.22)	1.80 (33.54)	−0.28	4.70
	Ling.-perc. bias	0.48 (0.66)	0.72 (65129.27)	−0.82	1.77
17	Intercept	2.24 (1.40)	1.60 (27.42)	−0.62	5.10
	Ling.-perc. bias	0.15 (0.69)	0.22 (66049.58)	−1.21	1.51
18	Intercept	2.20 (1.59)	1.39 (25.91)	−1.06	5.46
	Ling.-perc. bias	0.74 (0.72)	1.03 (66212.19)	−0.66	2.14
19	Intercept	0.75 (1.84)	0.41 (20.99)	−3.07	4.57
	Ling.-perc. bias	2.74 (0.73)	3.75 (65647.68)**	1.31	4.17
20	Intercept	0.38 (1.80)	0.21 (20.48)	−3.37	4.13
	Ling.-perc. bias	2.44 (0.70)	3.49 (65735.49)**	1.07	3.81

*Note*. Dependent variable is EEG activation: negative *t* values indicate a bias toward linguistic cortical areas, positive *t*-values a bias toward perceptual cortical areas; **p < 0.01, *p < 0.05.

**Table 3:** **Regression coefficients iconicity judgment EEG experiment**.

Time bin	variables	Estimate (SE)	*t* (d*f*)	CI lower	CI upper
1	Intercept	0.58 (1.23)	0.47 (27.25)	−1.94	3.09
	Ling.-perc. bias	−0.34 (0.54)	−0.63 (86498.15)	−1.41	0.72
2	Intercept	0.86 (1.36)	0.63 (27.96)	−1.92	3.64
	Ling.-perc. bias	−0.01 (0.55)	−0.02 (86822.04)	−1.09	1.07
3	Intercept	−0.07 (1.30)	−0.05 (29.12)	−2.73	2.59
	Ling.-perc. bias	2.14 (0.62)	3.47 (86759.75)**	0.93	3.34
4	Intercept	0.99 (1.41)	0.70 (28.46)	−1.89	3.87
	Ling.-perc. bias	−2.48 (0.61)	−4.10 (87120.52)**	−3.67	−1.29
5	Intercept	0.85 (2.03)	0.42 (21.39)	−3.37	5.06
	Ling.-perc. bias	−3.20 (0.60)	−5.37 (85757.91)**	−4.37	−2.03
6	Intercept	1.65 (1.89)	0.87 (23.25)	−2.25	5.56
	Ling.-perc. bias	−4.25 (0.57)	−7.43 (87672.08)**	−5.37	−3.13
7	Intercept	0.75 (1.88)	0.40 (22.18)	−3.14	4.64
	Ling.-perc. bias	−2.79 (0.55)	−5.03 (87008.84)**	−3.87	−1.70
8	Intercept	1.52 (1.11)	1.38 (46.84)	−0.71	3.76
	Ling.-perc. bias	−0.74 (0.54)	−1.36 (86591.37)	−1.80	0.33
9	Intercept	1.54 (1.43)	1.08 (25.22)	−1.40	4.48
	Ling.-perc. bias	−0.88 (0.49)	−1.79 (86759.15)	−1.84	0.08
10	Intercept	3.21 (1.20)	2.66 (29.05)*	0.75	5.67
	Ling.-perc. bias	−3.16 (0.52)	−6.11 (85320.16)**	−4.18	−2.15
11	Intercept	3.07 (0.94)	3.28 (61.11)**	1.20	4.94
	Ling.-perc. bias	−0.51 (0.49)	−1.03 (87746.70)	−1.47	0.46
12	Intercept	2.91 (1.72)	1.69 (22.63)	−0.65	6.47
	Ling.-perc. bias	1.28 (0.53)	2.43 (86582.36)*	0.25	2.31
13	Intercept	3.99 (2.18)	1.83 (20.53)	−0.55	8.52
	Ling.-perc. bias	−0.16 (0.53)	−0.30 (87051.02)	−1.21	0.89
14	Intercept	1.16 (1.07)	1.09 (50.98)	−0.98	3.31
	Ling.-perc. bias	0.49 (0.53)	0.93 (86359.82)	−0.54	1.52
15	Intercept	−0.36 (1.13)	−0.32 (54.91)	−2.63	1.90
	Ling.-perc. bias	1.71 (0.49)	3.48 (87276.97)**	0.75	2.67
16	Intercept	−0.87 (1.34)	−0.65 (27.95)	−3.60	1.87
	Ling.-perc. bias	3.60 (0.51)	7.03 (85655.56)**	2.59	4.60
17	Intercept	−3.17 (1.48)	−2.14 (25.29)*	−6.22	−0.13
	Ling.-perc. bias	4.89 (0.53)	9.24 (87200.29)**	3.85	5.93
18	Intercept	−4.84 (2.33)	−2.08 (19.25)	−9.71	0.04
	Ling.-perc. bias	4.46 (0.50)	8.90 (87181.78)**	3.48	5.45
19	Intercept	−4.17 (2.52)	-1.66 (18.41)	−9.45	1.11
	Ling.-perc. bias	3.64 (0.49)	7.39 (87015.12)**	2.67	4.61
20	Intercept	−2.80 (0.94)	−2.99 (41.90)**	−4.68	−0.91
	Ling.-perc. bias	5.51 (0.52)	10.62 (85437.82)**	4.49	6.52

*Note*. Dependent variable is EEG activation: negative *t* values indicate a bias toward linguistic cortical areas, positive *t*-values a bias toward perceptual cortical areas; **p < 0.01, *p < 0.05.

To further demonstrate the neurological evidence for relatively earlier linguistic processes and relatively later perceptual simulation, we fitted the *t*-test values for the 20 time bins using exponential, power law, and growth models. The fit of the sinusoidal curve was superior to these models across the two data conditions. Figure 3B presents the fit, the standard errors, and the values for the four variables. The sinusoidal fit converged in four iterations (iconicity task) and five iterations (semantic task) to a tolerance of 0.00001.

Using the sinusoidal model and the parameters derived from the data, the following figure emerged (Figure 3B). For both the semantic judgment and the iconicity judgment conditions, linguistic cortical regions dominated initially, followed later by perceptual cortical regions. As Figure 3B clearly shows, activation in linguistic cortical regions dominated in the semantic judgment task, whereas activation in perceptual cortical regions was prominent in the iconicity judgment task. Moreover, linguistic cortical regions showed greater activation relatively early in the trial, whereas perceptual cortical regions showed greater activation relatively late in processing. The results from these analyses are in line with results we obtained through both more commonly used source localization techniques and RT analyses, but they give a more detailed view of relative cortical activation for linguistic and perceptual processes throughout each trial.

## Discussion

The purpose of this experiment was to neurologically determine to what extent both linguistic and embodied explanations can be used in conceptual processing. The results of a semantic judgment and an iconicity judgment task demonstrated that both language statistics and perceptual simulation explain conceptual processing. Specifically, statistical linguistic frequencies best explain semantic judgment tasks, whereas iconicity ratings better explain iconicity judgment tasks. Our results also showed that linguistic cortical regions tended to be relatively more active overall during the semantic task, and perceptual cortical regions tended to be relatively more active during the iconicity task. Moreover, on any given trial, neural activation progressed from language processing cortical regions toward perceptual processing cortical regions. These findings support the conclusion that conceptual processing is both linguistic and embodied, both in early and late processing, however when comparing the relative effect of linguistic processes versus perceptual simulation processes, the former precedes the latter (see also Louwerse and Connell, 2011).

Standard EEG methods, such as ERP, are extremely valuable when identifying whether a difference in cortical activation can be obtained for different stimuli. The drawback of these traditional methods is that excessive stimulus repetition is required. Moreover, ERP is useful in identifying whether an anomaly is detected (Van Berkum et al., 1999) or whether a shift in perceptual simulation has taken place (Collins et al., 2011), but does not sufficiently answer the question to what extent different cortical regions are relatively more or less active than others. The technique shown here used source localization techniques to determine where differences in activation were present during early and late processing. We then used that information to compare the relative effect sizes of two clusters of cortical regions over the duration of the trial. This method is novel, yet its findings match those obtained from more traditional methods (Simmons et al., 2008; Louwerse and Jeuniaux, 2010; Louwerse and Connell, 2011). This method obviously does not render fMRI unnecessary for localization. In our analyses we compared the relative dominance of different clusters of cortical regions (filtering out their individual effects). Such a comparative technique does not allow for localization of specific regions of the brain; it only allows for a comparison of (predetermined) regions.

How can the findings reported in this paper be explained in terms of the cognitive mechanisms involved in language processing? We have argued elsewhere that language encodes perceptual relations (Louwerse, 2011). Speakers translate prelinguistic conceptual knowledge into linguistic conceptualizations, so that perceptual relations become encoded in language, with distributional language statistics building up as a function of language use (Louwerse, 2008). Louwerse (2007, 2011) proposed the Symbol Interdependency Hypothesis, which states that comprehension relies both on statistical linguistic processes as well as perceptual processes. Language users can ground linguistic units in perceptual experiences (embodied cognition), but through language statistics they can bootstrap meaning from linguistic units (symbolic cognition). Iconicity relations between words (Louwerse, 2008), the modality of a word (Louwerse and Connell, 2011), the valence of a word (Hutchinson and Louwerse, 2012), the social relations between individuals (Hutchinson et al., 2012), the relative location of body parts (Tillman et al., 2012), and even the relative geographical location of city words (Louwerse and Benesh, 2012) can be determined using language statistics. The meaning extracted through language statistics is, however, shallow, but provides good-enough representations. For a more precise understanding of a linguistic unit, perceptual simulation is needed (Louwerse and Connell, 2011). Depending on the stimulus (words or pictures; Louwerse and Jeuniaux, 2010), the cognitive task (Louwerse and Jeuniaux, 2010; current study), and the time of processing (Louwerse and Connell, 2011; current study) the relative effect of language statistics or perceptual simulations dominates. The findings reported in this paper support the Symbol Interdependency Hypothesis, with the relative effect of the linguistic system being more dominant in the early part of the trial and the relative effect of the perceptual system dominating later in the trial.

The RT and EEG findings reported here are relevant for a better understanding of the mechanisms involved in conceptual processing. They are also relevant for a philosophy of science. Recently, many studies have demonstrated that cognition is embodied, moving the symbolic and embodiment debate toward embodied cognition. The history of the debate (De Vega et al., 2008) is, however, reminiscent of the parable of the blind men and the elephant. In this tale, a group of blind men each touch a different part of an elephant in order to identify the animal, and when comparing their findings learn that they fundamentally disagree because they fail to see the whole picture. Evidence for embodied cognition is akin to identifying the tusk of the elephant, and evidence for symbolic cognition is similar to identifying its trunk. Dismissing or ignoring either explanation is reminiscent of the last lines of a parable: “For, quarreling, each to his view they cling. Such folk see only one side of a thing” (Udana, 6.4). Cognition is both symbolic and embodied; the important question now is under what conditions symbolic and embodied explanations best explain experimental data. The current study has provided RT and EEG evidence that both linguistic and perceptual simulation processes play a role in conceptual cognition, to different extents, depending on the cognitive task, with linguistic processes preceding perceptual simulation.

## Conflict of Interest Statement

The authors declare that the research was conducted in the absence of any commercial or financial relationships that could be construed as a potential conflict of interest.

## Appendix

### Experimental items used in Experiment 1 and 2

Airplane – runway

Antenna – radio

Antler – deer

Attic – basement

Belt – shoe

Billboard – highway

Boat – lake

Boot – heel

Bouquet – vase

Branch – root

Bridge – river

Car – road

Castle – moat

Ceiling – floor

Cork – bottle

Curtain – stage

Eyes – whiskers

Faucet – drain

Fender – tire

Flame – candle

Flower – stem

Foam – beer

Fountain – pool

Froth – coffee

Glass – coaster

Grill – charcoal

Handle – bucket

Hat – scarf

Head – foot

Headlight – bumper

Hiker – trail

Hood – engine

Icing – donut

Jam – toast

Jockey – horse

Kite – string

Knee – ankle

Lamp – table

Lid – cup

Lighthouse – beach

Mailbox – post

Mane – hoof

Mantle – fireplace

Mast – deck

Monitor – keyboard

Mustache – beard

Nose – mouth

Pan – stove

Pedestrian – sidewalk

Penthouse – lobby

Pitcher – mound

Plant – pot

Roof – porch

Runner – track

Saddle – stirrup

Seat – pedal

Sheet – mattress

Sky – ground

Smoke – chimney

Sprinkler – lawn

Steeple – church

Sweater – pants

Tractor – field

Train – railroad
